# Telomere fusion threshold identifies a poor prognostic subset of breast cancer patients

**DOI:** 10.1016/j.molonc.2015.02.003

**Published:** 2015-02-25

**Authors:** K. Simpson, R.E. Jones, J.W. Grimstead, R. Hills, C. Pepper, D.M. Baird

**Affiliations:** ^1^ Institute of Cancer & Genetics, School of Medicine, Cardiff University, Heath Park, Cardiff CF14 4XN, UK

**Keywords:** Breast cancer, Prognosis, Genome instability, Telomere

## Abstract

Telomere dysfunction and fusion can drive genomic instability and clonal evolution in human tumours, including breast cancer. Telomere length is a critical determinant of telomere function and has been evaluated as a prognostic marker in several tumour types, but it has yet to be used in the clinical setting. Here we show that high‐resolution telomere length analysis, together with a specific telomere fusion threshold, is highly prognostic for overall survival in a cohort of patients diagnosed with invasive ductal carcinoma of the breast (n = 120). The telomere fusion threshold defined a small subset of patients with an extremely poor clinical outcome, with a median survival of less than 12 months (HR = 21.4 (7.9–57.6), P < 0.0001). Furthermore, this telomere length threshold was independent of ER, PGR, HER2 status, NPI, or grade and was the dominant variable in multivariate analysis. We conclude that the fusogenic telomere length threshold provides a powerful, independent prognostic marker with clinical utility in breast cancer. Larger prospective studies are now required to determine the optimal way to incorporate high‐resolution telomere length analysis into multivariate prognostic algorithms for patients diagnosed with breast cancer.

## Highlights

- High‐resolution telomere length analysis is highly prognostic for overall survival.
- Independent of ER, PGR, HER2 status, NPI and grade.
- Dominant variable in multivariate analysis.

## 1 Introduction

The clinical management of breast cancer is informed by prognostic and predictive markers, which allow breast tumours to be classified into subtypes that can display distinct clinical outcomes. These markers include histopathological criteria such as tumour size, grade and lymph node metastasis: as well as the molecular markers, such as oestrogen receptor (ER) and progesterone receptor (PGR), that predict response to treatment with endocrine therapy, or human epidermal growth factor receptor 2 (HER2) that predicts response to HER2 antagonists (Subramaniam and Isaacs, 2005). These data are used in combination with multivariate algorithms to inform on treatment options and to provide prognostic information (Mook et al., 2009). These markers can be augmented by gene expression profiling that has allowed the identification of additional molecular subtypes, which can inform about treatment options in specific clinical subsets (Hennessy et al., 2009). Whilst these markers can inform the clinical management of breast cancer, they are unable to provide definitive individualised prognostic information.

Whole genome sequence analysis of breast tumours has revealed further levels of genetic heterogeneity that can impact on clinical outcome, it is apparent from these studies that increasing genomic complexity confers a poorer prognosis (Curtis et al., 2012). The underlying mechanisms driving genomic instability and selection for complex genomes in breast cancer is not entirely clear, but likely includes DNA repair and checkpoint defects, as well as telomere dysfunction (Kwei et al., 2010). Telomeres are structures that cap the ends of chromosomes and prevent aberrant repair of the natural chromosome end by the DNA double strand break repair apparatus (de Lange, 2005). Telomere erosion, as a function of cell division, can lead to the formation of short dysfunctional telomeres that have lost the end‐capping function and are capable of fusion with other telomeres or non‐telomeric loci (Capper et al., 2007; Counter et al., 1992). The presence of short dysfunctional telomeres, which are capable of fusion and associated with genomic complexity, have been detected in many tumour types, including breast cancer (Chin et al., 2004; Lin et al., 2010; Roger et al., 2013). These data are consistent with tumour cells undergoing a period of ‘telomere crisis’ during the progression to malignancy, which drives large‐genomic rearrangements and creates the diversity on which clonal selection can operate. Telomere dysfunction and fusion may represent a common mechanism of genomic instability facilitating progression in a broad range of tumour types.

Importantly, short dysfunctional telomeres have been detected in early‐stage tumours (Lin et al., 2010; Meeker et al., 2004b), as well as in premalignant lesions. This indicates that telomere dysfunction can precede disease progression and is not simply a biomarker of advanced disease. Indeed, recent evidence suggests that short telomeres may be an inherent property of a proportion of cells in which the tumour initiating event occurred (Roger et al., 2013). Telomere length in early stage lesions varies considerably between different tumour clones, leading to the hypothesis that clones harbouring short dysfunctional telomeres exhibit a mutator phenotype that can drive clonal evolution and progression: whereas those exhibiting longer telomeres, may have a more stable genome and be less prone to clonal evolution (Roger et al., 2013). Thus, by informing on the propensity of a tumour to undergo clonal evolution, the telomere length of early‐stage tumours, has the potential to provide prognostic information. We have recently examined this hypothesis in chronic lymphocytic leukaemia (CLL), in which we had previously identified that telomere shortening and dysfunction can occur prior to clinical progression (Lin et al., 2010). We established the telomere length threshold below which telomere fusion could be detected and we used this to stratify patients based on telomere length (Lin et al., 2014). These data show that telomere lengths below the fusion threshold are highly prognostic and that the mean of the fusogenic range (2.26 kb) provided the optimum prognostic resolution. Indeed, telomere length below the fusion threshold was the most powerful predictor of survival in CLL and this was particularly prognostic in early‐stage patients (HR = 19.3 (17.8–802.5), P < 0.0001) (Lin et al., 2014). Given the prognostic significance of our findings in CLL and that telomere dysfunction has been implicated in many tumour types, we wanted to establish whether our threshold for telomere dysfunction could predict outcome in other common solid human cancers. Here we show that the telomere fusion threshold, established in CLL, identifies a subgroup of breast cancer patients with an extremely poor clinical outcome.

## 2 Materials and methods

### 2.1 Breast cancer cohort

DNA samples extracted from frozen tissue blocks obtained from surgically resected Invasive Ductal carcinoma of the breast were obtained with ethical approval from the Wales Cancer Bank. A certified histopathologist determined percentage tumour in each section as detailed in Supplementary Table 1. DNA extraction was performed using the Qiagen TissueLyser to homogenize the tissue and the QIAamp DNA mini kit (Qiagen, Cat#: 51306) according to the manufacturers guidelines. The median follow‐up of the cohort was 4.6 years and the patients' clinical/laboratory characteristics are summarized in Table 1 and provided in full in Supplementary table 1.

**Table 1:** Clinical characteristics of the invasive ductal breast carcinoma cohort (n = 120).

Factor	Subset	Number
Median age		60.5 years
Range		33–87 years
Median follow‐up		4.6 years
Grade	I	11
	II	47
	III	62
ER status	negative	30
	positive	89
	not determined	1
PGR status	negative	36
	positive	41
	not determined	43
NPI status	<3.4	14
	>3.4/<5.4	37
	>5.4	18
	not determined	51
HER2 status	negative	78
	positive	27
	not determined	15
Adjuvant chemotherapy	negative	39
	positive	81
Adjuvant radiotherapy	negative	31
	positive	89
Adjuvant hormone therapy	negative	26
	positive	91
	unknown	3

ER = estrogen receptor.

PGR = progesterone receptor.

NPI = Nottingham Prognostic Index.

HER2 = human epidermal growth factor receptor 2.

### 2.2 Telomere length analysis

For telomere length analysis at the XpYp telomere we used the single telomere length analysis (STELA) assay as previously described (Baird et al., 2003; Capper et al., 2007); for three samples that displayed bimodal telomere length distributions, we used the mean of lower modal distribution.

### 2.3 Statistical methods

Statistical analysis including the Mann–Whitney and Chi squared tests were carried out using Prism 6.0 (Graphpad), SPSS version 20 (IBM) and SAS version 9.3 software (SAS Institute). Univariate comparisons for overall survival (OS) were conducted with the log‐rank test, displayed as Kaplan Meier curves and hazard ratios calculated using the cox proportional hazards model. Analyses of time to event outcomes with respect to continuous variables or those with less than two categories, together with multivariable analyses, were performed using a Cox proportional hazard model with forward selection. P < 0.05 was considered significant. Hazard Ratio (HR).

## 3 Results

**Figure 1:**
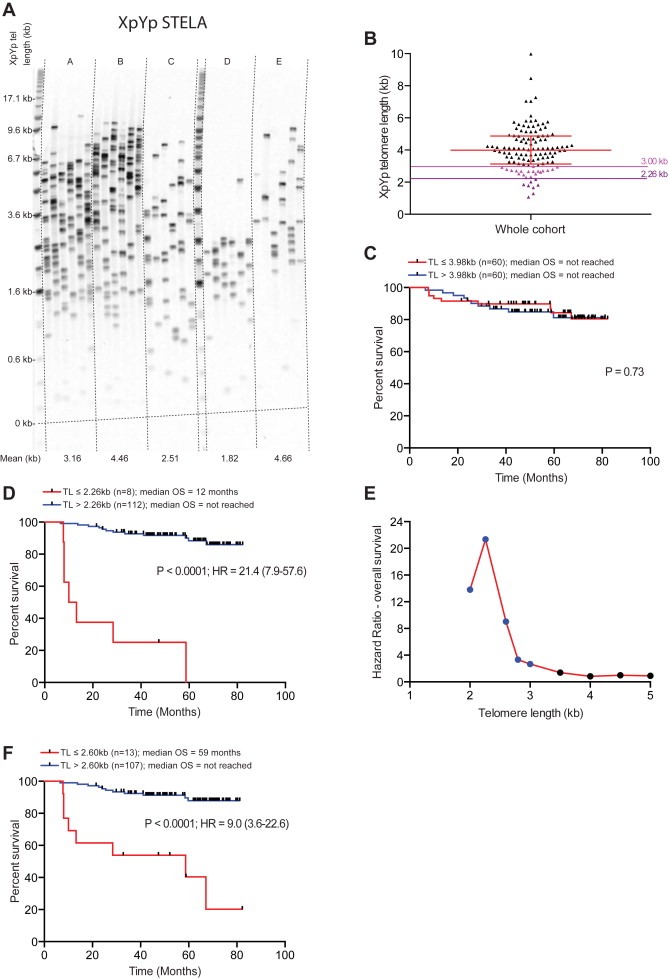
Telomere dysfunction is highly prognostic in breast cancer. (A) An example of XpYp STELA in 5 breast cancer samples. (B) Mean telomere length in a cohort of patients diagnosed with ductal breast carcinoma (n = 120), telomere length was determined using single telomere length analysis (STELA). The telomere length thresholds that provide prognostic information are indicated with coloured lines, the upper limit 3 kb (pink) and the optimum limit of 2.26 kb (purple). (C) Kaplan Meier curve for overall survival (OS) using the median telomere length of the cohort to stratify patients. Telomere Length (TL) used to stratify patients, P value, Hazard Ratio (HR) and 95% confidence interval are indicated on the plots together with numbers at risk in each arm (N). (D) Kaplan Meier curve for overall survival using a telomere length of 2.26 kb to stratify patients. (E) Recursive partitioning of mean telomere length in the same cohort and plots the hazard ratios for overall survival for each threshold for the entire cohort; 2.26 kb provided the optimal discrimination in this breast cancer cohort. Blue markers indicate significant HRs (P < 0.05), black markers indicate non‐significant differences (P > 0.05). (F) Kaplan Meier curve for overall survival using a telomere length of 2.60 kb to stratify patients.

We performed high‐resolution single telomere length analysis (STELA) on DNA obtained from 120 tumour samples from patients diagnosed with invasive ductal carcinoma of the breast with a median follow‐up of 4.6 years (Table 1). STELA is capable of detecting the full spectrum of telomere lengths at specific chromosome ends (Baird et al., 2003), of particular importance is its ability to detect the presence of short telomeres within the length ranges at telomeres are capable of fusion (Figure 1A; (Capper et al., 2007). Consistent with previous reports, telomere length of the tumour samples was not associated with age (r^2^ = 0.009) (Martinez‐Delgado et al., 2013). There was marked heterogeneity in the mean telomere length distributions between patients ranging from 1.07 kb to 9.97 kb (median 3.98 kb). 8/120 (7%) of the cohort displayed telomere length distributions of less than the 2.26 kb fusion threshold (Figure 1B). Stratification of the cohort based on the median telomere length provided no prognostic resolution (P = 0.73; Figure 1C). In contrast, the optimum fusion threshold (2.26 kb), identified in CLL, was a strong predictor of overall survival (P < 0.0001; HR = 21.4 (7.9–57.6), Figure 1D). Patients with telomere lengths below the threshold displayed a median survival of 348 days (95% CI 233–1760) whereas patients above the threshold showed 89% (95% CI 80%–94%) survival at 60 months.

Recursive partitioning based on incremental telomere length thresholds revealed that the 2.26 kb threshold provided the optimal prognostic resolution (Figure 1E). However, it was also apparent that longer telomere length thresholds, within the fusogenic range, were also highly prognostic (Figure 1E). For example 11% of the cohort display mean telomere length profiles of less than 2.6 kb; this threshold was also highly predictive of clinical outcome (P < 0.0001; HR = 9.0 (3.6–22.6); Figure 1F). The longest telomere length that still provided significant prognostic resolution was 3 kb, which equated to the lower quartile of telomere length distributions within the cohort (P < 0·035; HR = 2.7 (1.1–6.7); Figure 1B and E).

**Figure 2:**
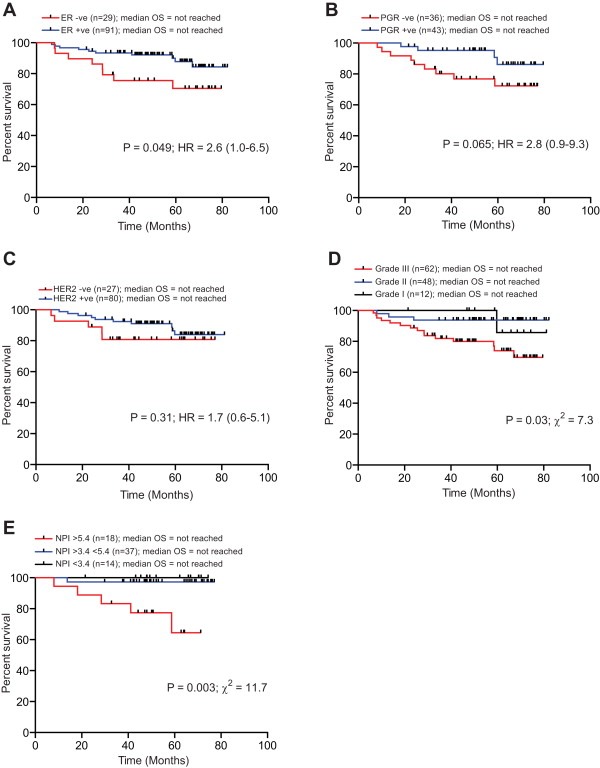
Telomere dysfunction provides increased prognostic resolution for overall survival compared to the commonly used markers in breast cancer. Kaplan Meier curves for overall survival of the same cohort of patients diagnosed with ductal breast carcinoma. (A) estrogen receptor (ER) to stratify patients. (B) Progesterone receptor (PGR). (C) Human epidermal growth factor receptor 2 (HER2). (D) histological grade. (E) Nottingham Prognostic Index (NPI). P value, Hazard Ratio (HR), Chi Squared value (χ2) and 95% confidence interval are indicated on the plots together with numbers at risk in each arm (N).

**Figure 3:**
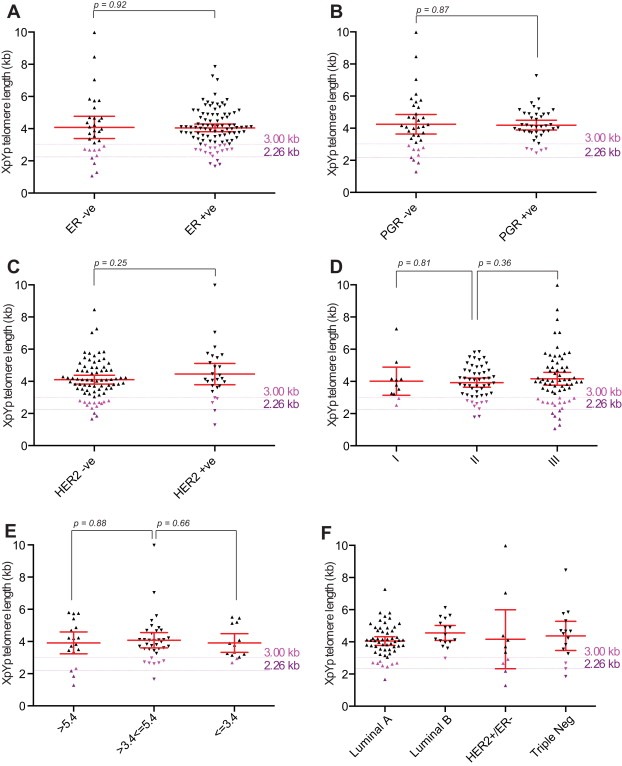
Telomere length in breast cancer is independent of ER, PGR, HER2 status, NPI score and grade. (A) Scatter plot displaying mean XpYp telomere length of ER positive or negative breast cancers as indicated. P value derived from a Mann–Whitney test is displayed above. The telomere length thresholds that provide prognostic information are indicated with coloured lines, the upper limit 3 kb (pink) and the optimum limit of 2.26 kb (purple). (B) Scatter plot displaying mean XpYp telomere length of PGR positive or negative breast cancers as indicated. (C) Scatter plot displaying mean XpYp telomere length of HER2 positive or negative breast cancers as indicated. (D) Scatter plot displaying mean XpYp telomere length according to grade. (E) Scatter plot displaying mean XpYp telomere length according to NPI score. (F) Scatter plot displaying mean XpYp telomere length according to molecular sub‐type, Luminal A (ER+ and/or PR+, HER2−), Luminal B (ER+ and/or PR+, HER2+), HER2+/ER− (ER−, PR−, and HER2+) and Triple Negative (ER−, PR−, HER2−).

The fusion threshold was significantly more prognostic than the established clinical and laboratory markers evaluated in the same cohort (Figure 2). No relationship was detected between telomere length and the status of the commonly used prognostic markers (Figure 3A–D). Furthermore, patients with short telomeres (<3.0 kb) were observed in all of the established prognostic subsets, including estrogen receptor (ER), progesterone receptor (PGR) and human epidermal growth factor receptor 2 (HER2) positive and negative subsets (Figure 3A–C), as well as in all 3 histopathologic grades (Figure 3D) and NPI score (Figure 3E). Categorisation of breast tumours into sub‐types based on ER, PGR, HER2 expression patterns revealed no statistically significant differences in the overall telomere length distributions or the proportions of tumours with telomere lengths within the length range that provides prognostic significance (Figure 3F). These data indicate that the poor prognosis of patients with tumours exhibiting short telomeres is independent of the established markers used in breast cancer. This was confirmed in multivariate analysis using Cox proportional hazards with forward selection. The multivariate analysis showed that, despite the relevantly small number of patients fully characterised for all the established markers (Table 1), the telomere fusogenic mean was the most significant parameter for survival (P < 0.0001; HR = 8.1 (6.2–12.7)); no other parameters met the P < 0.05 level for entry into the model including age, Nottingham prognostic index, oestrogen receptor status, progesterone receptor status, tumour grade and HER2 status. These data indicate the potential of high‐resolution telomere length analysis for the stratification of patients with breast cancer; a larger validation cohort is required to substantiate these findings.

## 4 Discussion

The underlying molecular mechanisms that result in the large‐scale genomic aberrations observed in some breast cancer subtypes are not clear (Kwei et al., 2010); however there is evidence to implicate telomere dysfunction in this process. In common with many solid tumours, telomere erosion has been documented in breast cancer (Odagiri et al., 1994) and evidence of telomere dysfunction has been observed early in the progression of the disease (Chin et al., 2004; Meeker and Argani, 2004; Tanaka et al., 2012). Furthermore, copy number aberrations, of the type generated by telomere‐dysfunction, confer a poorer prognosis (Curtis et al., 2012). Given this, we set out to use the telomere length thresholds below which telomere fusion is detected in CLL, together with high‐resolution telomere length analysis, in a retrospective study of tumour DNA from a cohort of breast cancer patients with invasive ductal carcinoma (n = 120). Our data show that a small subset of breast tumours exhibit telomere length profiles within the length ranges in which telomere fusion can occur and these patients display a poor prognosis. These data are consistent with the view that the progression to malignancy in breast cancer requires transition through a telomere‐driven crisis and that this facilitates clonal evolution and progression.

Telomere length has previously been assessed as a prognostic marker in breast cancer. Lu et al. used high throughput qPCR in a cohort of breast tumours and found no association with any of the clinical or pathological features of the disease (Lu et al., 2011). Whereas a previous study determining telomere content, not length, using slot blots showed that the lower tertile of telomere content was prognostic for overall survival (Fordyce et al., 2006; Heaphy et al., 2007). Consistent with the Lu et al. study, our data showed no prognostic significance when using the median telomere length of the cohort as the cut off for long or short telomeres (Lu et al., 2011). However, when using the same telomere length threshold previously defined in CLL to stratify the patients, we demonstrated remarkable prognostic discrimination for overall survival in breast cancer patients. A small subset of patients with short telomeres within the telomere fusogenic range had a median survival of 12 months with no patients surviving beyond five years. In contrast, 88% of patients with telomeres above the fusogenic range were alive at five years. The fusogenic threshold was independent of the commonly used markers in breast cancer and was the dominant variable in multivariate analysis. This study was limited in both in the size of the cohort and the length of clinical follow up data available and clearly our findings will require validation in a larger cohort with a longer clinical follow‐up. However these data do indicate that high‐resolution telomere length analysis, coupled with the telomere fusion threshold, identifies a subset of patients a particularly poor prognosis. This group of patients could not be identified with the commonly used prognostic markers in breast cancer. Taken together our data suggest that high‐resolution telomere length analysis could be a useful component in multivariate prognostic algorithms.

Our data show that telomere shortening can be extensive in breast tumours, yet this appears to occur in the presence of telomerase, which is detected in the majority of breast tumours analysed (Hiyama et al., 1996; Pearson et al., 1998). However an absence of any relationship between the levels of telomerase activity and telomere length has been reported in Breast cancer (Lu et al., 2011) and other tumour types (Wang et al., 2002). We have recently shown that short telomeres can be detected in early stage lesions for example in colorectal adenomatous polyps (Roger et al., 2013) and in chronic lymphocytic leukaemia prior to clinical progression (Lin et al., 2010, 2014). Importantly these data also show that short telomeres, of the same length as that observed tumours, can also be detected within the telomere length profiles of normal tissue. These observations have allowed us to conclude that the majority of telomere erosion may occur in the normal tissue prior to the initiating event. This may also be the situation in normal breast epithelium; consistent with this view, *in situ* hybridization has revealed telomere shortening in subsets of normal breast epithelium (Meeker et al., 2004a).

In addition to providing prognostic resolution, our telomere‐length data imply that telomere dysfunction is important in the pathogenesis of breast cancer, a view that is consistent with data from other laboratories (Chin et al., 2004; Tanaka et al., 2012). Moreover BRCA1/2 breast tumours with short telomeres have also been associated with higher levels of apoptotic markers and p53 overexpression, consistent with DNA repair defects and genomic instability (Martinez‐Delgado et al., 2013). Telomere fusion arises as consequence of aberrant DNA repair activities at short telomeres and there is growing body of evidence to implicate alternative non‐homologous end joining (A‐NHEJ) in this process (Letsolo et al., 2010; Rai et al., 2010; Tankimanova et al., 2012). Poly [ADP‐ribose] polymerase 1 (PARP1), Ligase III and to lesser extent Ligase I have been implicated in A‐NHEJ (Boboila et al., 2012; Simsek et al., 2011), these proteins, in particular PARP1, have provided potential therapeutic targets (Chen et al., 2008; Rouleau et al., 2010). Our data indicate the intriguing possibility that breast tumours, exhibiting telomeres within the length range at which fusion is detected, may be sensitive to agents targeted to the A‐NHEJ pathway.

Telomere dysfunction has been implicated in the progression of numerous tumour types (Svenson and Roos, 2009), we therefore considered that the telomeric parameters defined in CLL may be applicable to other tumour types. Here we show that this is the case for breast cancer and therefore our data pinpoint an important biological limit threshold for telomere length in both CLL and breast tumours; patients with telomeres below the telomere fusogenic mean exhibited a consistently inferior clinical outcome. We therefore consider that the telomere fusion threshold will be applicable to a broad spectrum of tumour types.

## Funding

This work was supported by Cancer Research UK (C17199/A13490). CP and DMB are also supported by the National Institute for Social Care and Health Research (NISCHR) through the Cancer Genetics Biomedical Research Unit.

## Disclosure

DMB and CP declare co‐authorship of a patent application based on some of this work. All remaining authors have declared no conflicts of interest.

## Supporting information



The following is the supplementary data related to this article:

Supplementary data

## Supplementary data 1

### 1.1

Supplementary data related to this article can be found at http://dx.doi.org/10.1016/j.molonc.2015.02.003.
