# Supplementary material for: Telomere fusion threshold identifies a poor prognostic subset of breast cancer patients
Source: Mol Oncol. 2015 Feb 25;9(6):1186–93. doi: 10.1016/j.molonc.2015.02.003 (PMC4449122; doi:10.1016/j.molonc.2015.02.003)
Supplement: Supplementary file 1 — Supplementary data [file MOL2-9-1186-s001.pdf]

Supplemental table 1. Displaying the telomere length data, together with the clinical/laboratory characteristics for each patient with Invasive Ductal carcinoma.

Sample number	Age at Operation	Percent Tumor	pTNM	NPI Score	Breast Grade	ER	PGR	HER2	Alive	OS	Telomere length
1	84	81-100%		0	III	Negative			1	233	1.07
2	75	81-100%	T2 N3a Mx	6.82	III	Negative	Negative	positive	1	852	1.29
3	68	81-100%	T1c No Mx	4.36	III	positive		negative	0	1423	1.66
4	82	81-100%	T2 No Mx	0	II	positive			1	241	1.78
5	69	81-100%		0	II				1	396	1.82
6	43	81-100%	T2 N2a Mx	6.46	III	Negative	Negative	negative	1	1760	1.84
7	72	81-100%	T2 N1 Mx	0	III	positive	Negative	negative	1	300	2.02
8	79	81-100%	T2N1miMx	5.42	III	Negative	Negative	positive	1	241	2.18
9	61	81-100%	T1 No Mx	0	II	positive			0	2467	2.29
10	73	81-100%	T2N1Mx	5.84	III	Negative	Negative	negative	0	985	2.33
11	59	81-100%		0	II	positive	positive	negative	0	1564	2.46
12	75	81-100%	T2 N1 Mx	0	III	positive			1	2015	2.51
13	79	81-100%	T1 No	0	I	positive		negative	0	1766	2.52
14	45	81-100%	T2N1aMx	4.8	II	positive	positive	negative	0	1997	2.62
15	59	81-100%	T2N0Mx	4.44	III	positive	positive	negative	0	1477	2.66
16	73	81-100%	T2NOMX	4.72	III	Negative	Negative	positive	0	1265	2.67
17	48	21-40%	T1 No Mx	0	III	Negative	Negative	negative	0	2305	2.68
18	63	81-100%	T1c No Mx	3.28	II	positive		negative	0	2006	2.69
19	34	81-100%	T2 No Mx	0	III	Negative	positive		0	2388	2.70
20	65	61-80%		0	II	positive		negative	0	1570	2.70
21	65	81-100%	T1c No Mx	4.36	III	Negative	positive	negative	0	1973	2.73
22	56	81-100%	T2 No Mx	0	II	positive			0	2407	2.73
23	53	41-60%	T2mN1Mx	4.54	II	positive		negative	0	1128	2.79
24	58	81-100%	T2 No Mx	0	III	positive	Negative	negative	0	2271	2.80
25	58	81-100%	T2 No Mx	0	III	Negative	Negative	positive	0	2264	2.92
26	83	61-80%	T1cNoMx	2.4	I	positive			0	2142	2.95
27	38	81-100%		4.3	III	positive		positive	0	2179	2.98
28	74	81-100%	T1cNoMx	3.34	II	positive			0	2109	3.04
29	75	81-100%	t1n0mx	0	II	positive	positive	negative	0	1493	3.04
30	73	81-100%	T2 N1 Mx	0	II	Negative	Negative		0	2306	3.12
31	47	81-100%	T2N0Mx	3.4	II	positive		negative	0	1442	3.15
32	86	81-100%		0	II	positive			0	2432	3.16
33	81	41-60%	T2NoMx	2.6	I	positive	positive	negative	0	646	3.22
34	42	81-100%	T1c No Mx	2.24	I	positive			0	1977	3.26
35	39	81-100%	T2N0Mx	4.6	III	Negative	Negative	negative	0	2148	3.26
36	45	81-100%	T2 N1	0	II	positive		negative	0	2060	3.26
37	50	41-60%	T1cNoMx	3.28	II	positive		negative	0	1295	3.35
38	42	81-100%	T2N3aMx	6.46	III	Negative	Negative	positive	0	1329	3.37
39	66	81-100%	T1c N1 Mx	5.26	III	positive	positive	negative	0	2022	3.43
40	78		T2 N1 Mx	5.48	III	positive			0	1927	3.45
41	47	81-100%	T2 N1m Mx	5.6	III	positive		negative	0	1917	3.50
42	65	21-40%	T2N1aMx	3.56	I	positive		negative	0	1408	3.52
43	73	61-80%	T2 No Mx	3.56	II	Negative	Negative	negative	0	2077	3.54
44	58	81-100%	T3 N1 Mx	5.02	II	positive	positive	negative	0	2228	3.55
45	59	81-100%	T2 No Mx	0	III	positive	Negative	negative	0	2141	3.58
46	79	81-100%	T2N0Mx	4.66	III	positive		positive	0	1492	3.60
47	69	81-100%	T1c No Mx	4.34	III	Negative		positive	0	1922	3.70
48	61	81-100%	T3N1miMx	5.08	II	positive		negative	0	1231	3.70
49	68	81-100%	T2 N1a Mx	5.46	III	positive	positive	negative	0	2137	3.72
50	36	41-60%		0	II	positive	Negative	positive	0	1749	3.72
51	60	81-100%	T2 N12 Mx	4.54	II	positive	positive	negative	0	2268	3.75
52	69	61-80%		0	II	positive	positive	negative	0	1935	3.76
53	45	61-80%	T1c No Mo	2.28	I	positive	positive	negative	0	2233	3.78
54	68	81-100%	T2aNoMx	3.44	II	positive	positive	negative	0	1563	3.81
55	52	81-100%		0	III	positive	positive	negative	1	1754	3.82
56	49	81-100%	T2N1miMx	5.52	III	positive		negative	0	1414	3.83
57	46	81-100%	pT2 No Mx	4.6	III	Negative	Negative	negative	0	2221	3.83
58	70	81-100%	T4bNoMx	3.28	II	positive	positive	negative	0	1479	3.89
59	83	81-100%	T2N0MX	0	III	positive	positive	positive	0	723	3.96
60	55		T1cN0Mx	4.36	III	positive	positive	negative	0	896	3.98
61	48	81-100%	t2n0mx	0	III	positive	Negative	positive	0	721	3.99
62	68	61-80%		0	III	positive	positive	negative	1	766	4.02
63	87	81-100%	T2NoMx	4.66	III	positive	Negative	positive	0	2157	4.06
64	70	81-100%	T1cN0Mx	3.3	II	positive	positive	negative	0	1362	4.08
65	70	81-100%	T2 No Mx	4.42	III	positive	Negative	negative	0	1948	4.08
66	53	81-100%	T2 N1 Mx	0	II	positive		negative	0	1725	4.09
67	46	81-100%	T2NoMx	3.96	I	positive	positive	negative	0	1436	4.10
68	58	81-100%	T2 N1a Mx	4.44	II	positive		negative	0	2282	4.11
69	71	81-100%	T2 No Mx	4.44	III	Negative	Negative	positive	0	2313	4.11
70	51	81-100%	T2 N2 Mx	0	III	positive		positive	1	195	4.12
71	48	41-60%	T2N0Mx	4.44	III	positive	positive	negative	0	1142	4.12
72	80	81-100%		0	I	positive	positive	negative	1	1792	4.15
73	49	81-100%	T2 No Mx	0	I	positive		negative	0	2436	4.16
74	56	81-100%	T1N0MX	0	II	positive	Negative	positive	0	1048	4.17
75	47	81-100%	T2N2aMx	5.98	II	positive	positive	negative	0	1508	4.20
76	61	81-100%	T1cN1aMx	4.24	II	positive	positive	negative	0	1638	4.22
77	76	61-80%	T2mN3aMx	6.8	III	positive	positive	negative	0	1502	4.22
78	33	81-100%	T2N1Mx	5.76	III	Negative	Negative	negative	0	1523	4.23
79	67	81-100%	T2 N1 Mx	0	II	positive		negative	0	2173	4.29
80	48	81-100%		0	III	Negative		positive	1	854	4.38
81	58	81-100%	T1 No	0	II	positive		positive	0	1907	4.46
82	65	81-100%	T2N0Mx	4.44	III	Negative	Negative	negative	0	1442	4.50
83	86	41-60%		0	II	positive	positive	negative	0	1625	4.55
84	40	81-100%	T1 No Mx	0	II	positive	positive	negative	0	2271	4.63
85	54	81-100%	T1c No Mx	4.32	III	Negative	Negative	negative	0	2232	4.66
86	42	61-80%	T3N0MX	0	III	Negative	Negative	negative	1	1000	4.66
87	49	61-80%	T2 No Mx	3.44	II	positive		negative	0	1527	4.69
88	60	81-100%	T2N1aMx	5.62	III	Negative	Negative	negative	0	1440	4.70

89	48	81-100%	T2N1aMx	4.6	II	positive	positive	negative	0	1589	4.70
90	48	81-100%	T1 N1 Mo	5.34	III	positive	positive	negative	0	2286	4.87
91	50	81-100%	T2N1Mx	5.66	III	positive	positive	negative	1	546	4.87
92	70	81-100%	T1 No Mx	0	II	positive	positive	negative	0	2095	4.89
93	46	81-100%	T2N0MX	0	III	positive	positive	positive	0	1085	4.89
94	53	41-60%	T2	0	III	positive	Negative	positive	0	1858	4.90
95	72	41-60%	Unknown	0	II	positive	positive	negative	0	1935	4.93
96	60	81-100%	T2NoMx	3.44	II	positive			0	1274	4.99
97	65	81-100%	T1cN0Mx	3.24	II	positive		negative	0	1439	5.14
98	69	81-100%		0	II	positive	positive	negative	0	2131	5.15
99	43	81-100%	t2n1m0	0	III	positive	positive	positive	0	1281	5.15
100	87	81-100%	T2NoMx	2.44	I	positive	positive	negative	0	1866	5.20
101	70	81-100%	T2N0Mx	4.82	III	Negative	Negative	negative	1	412	5.30
102	43	81-100%		0	II	positive	positive	negative	0	1913	5.35
103	76	61-80%	T2 N3a Mx	6.64	III	positive	Negative	negative	1	1233	5.42
104	42	81-100%	T1N0Mx	3.26	II	positive		positive	0	1561	5.46
105	66	81-100%		0	II	positive		negative	0	1884	5.48
106	84	81-100%	T1c No Mx	3.4	II	positive			0	2113	5.52
107	42	81-100%	T2N1biMO	0	III	positive	positive	positive	0	959	5.58
108	73	81-100%	T2N0Mx	4.28	III	positive		negative	0	1307	5.60
109	44	81-100%	T1cN1biMX	0	III	positive	Negative	positive	0	1071	5.64
110	66	81-100%	T2 N1 Mx	5.7	III	Borderline	Borderline	positive	0	1885	5.74
111	49		T2 N1 Mx	6	III	Negative	Negative	negative	0	1921	5.75
112	52	81-100%	T2N3aMx	6.58	III	positive		negative	0	1328	5.80
113	68	0-20%		0	II	positive	positive	negative	0	2122	5.81
114	71	81-100%	T2 N1 Mx	0	II	Negative	Negative	negative	1	719	5.85
115	75	81-100%		0	III	positive	Negative	positive	1	678	6.13
116	78	81-100%	T4bNoMx	4.5	III	positive	Borderline	negative	0	1344	7.04
117	45	81-100%	T3N2Mx	0	III	Negative	Negative	positive	0	991	7.05
118	67	41-60%	Unknown	0	I	positive	positive	negative	0	1507	7.27
119	47	61-80%	T1 No Mx	0	III	Negative	Negative	negative	0	2145	8.46
120	55	81-100%	T2 No Mx	4.44	III	Negative	Negative	positive	0	2058	9.97
